# Management of iatrogenic rectal injury following radical prostatectomy: a 10-year experience from a regional Australian hospital and an algorithm for management

**DOI:** 10.3389/fsurg.2026.1906216

**Published:** 2026-08-12

**Authors:** Khang Duy Ricky Le, Kevin Kah Wai Yoong, Leon Yeung, Iman Hameed, Nicholas Ang, David Patrick Heath, David Lloyd, Minh Hung Nguyen

**Affiliations:** 1 Department of Surgery, Launceston General Hospital, Launceston, TAS, Australia; 2 School of Medicine, Faculty of Health, Deakin University, Geelong, VIC, Australia; 3 Department of Medical Education, Faculty of Medicine, Dentistry and Health Sciences, University of Melbourne, Melbourne, VIC, Australia

**Keywords:** colorectal surgery, postoperative complications, prostatectomy, rectal fistula, rectal injury, rectourethral fistula.

## Abstract

**Background:**

Iatrogenic rectal injury following radical prostatectomy or cystoprostatectomy is a rare but significant complication associated with significant morbidity, including sepsis, rectourethral or rectovesical fistulas, and death. The management of these injuries remains highly heterogeneous, particularly in regional contexts where surgical rescue expertise is varied. This case series explores a decade of regional Australian experience in the management of rectal injuries following radical prostatectomy and proposes a practical management algorithm for multidisciplinary decision-making.

**Methods:**

A retrospective consecutive case series was conducted at a regional Australian tertiary hospital. Adult patients diagnosed with an iatrogenic rectal injury following a radical prostatectomy between January 2015 and December 2025 were identified. Demographic, operative, diagnostic, management, and outcome data were collected and analysed descriptively.

**Results:**

Eight patients were included. Rectal injuries were identified intraoperatively in three patients (37.5%) and postoperatively in five (62.5%), with delayed presentation ranging from 5 days to 2.5 years. Injury size ranged from <1 to 4 cm, and all defects involved a full-thickness rectal wall injury. Management strategies varied according to the timing of diagnosis, defect characteristics, tissue quality, and fistula formation. Immediate injuries were successfully managed with primary repair, with or without faecal diversion. Three patients developed rectourethral or rectovesical fistulas, requiring staged reconstruction using a transperineal approach with interposition of a pedicled gracilis flap. All patients achieved successful healing without mortality, and all diverting stomas were ultimately reversed.

**Conclusions:**

Rectal injury following prostatectomy requires prompt recognition and individualised multidisciplinary management. Early diagnosis facilitates simpler repair strategies, whereas delayed recognition is associated with fistula formation and the need for complex reconstructive procedures.

## Introduction

Prostate cancer is estimated to be the third most common cancer and the third leading cause of cancer-related mortality worldwide (1). Radical prostatectomy remains a cornerstone of curative-intent treatment whereby the entire prostate gland is surgically removed. Long-term data suggest that radical prostatectomy yields significant benefit for patients, with excellent control of localised disease and a significant improvement in oncological survival (2). Furthermore, the advent of minimally invasive approaches to radical prostatectomy, such as with robotic assistance, has led to improved outcomes related to length of stay and intraoperative bleeding (3, 4).

Rectal injury during prostatectomies remains a rare but severe complication with an estimated incidence of 0.1%–1.8% of patients (5, 6). When unidentified or identified late, it can confer significant morbidity, such as through the development of wounds or deeper pelvic infections or abscesses, rectal fistulas (such as to the urethra or anastomosis), sepsis, and even death (7). Many studies have explored risk factors related to the development of these injuries, which include prior prostatic or pelvic radiotherapy, undergoing prostatectomy at low-volume centres, and the extent of the prostate tumour (grade and stage) (5, 8, 9). Despite this, the best approach for the management of rectal injuries remains highly heterogeneous, influenced by factors such as surgical expertise, the nature of the injury, and the aforementioned patient risk factors. In essence, when identified intraoperatively, layered closure with or without diversion may be considered (5). However, the timing of and approach to the repair and the best outcomes of different management approaches remain poorly characterised, particularly in regional or rural settings where specialist colorectal expertise may not be available.

This study presents our 10-year experience with rectal injuries following radical prostatectomy, including the identification of the determinants of these types of injuries and our multidisciplinary approach to management. In doing so, we propose an algorithm for management and considerations for the regional and rural workforces to ensure they remain well-equipped to manage this important complication against a background of an ever-increasing volume of radical prostatectomies performed in non-metropolitan centres.

## Methods

### Study design

This retrospective study involved a consecutive case series of adult patients aged > 18 years who underwent radical prostatectomies with subsequent iatrogenic rectal injury and were diagnosed and managed at Launceston General Hospital, a 308-bed tertiary-level regional public hospital in Launceston, Tasmania, Australia. Rectal injury was defined as a breach of any or all of the layers of the rectal wall, defined radiologically, endoscopically, or intraoperatively, following index radical prostatectomy, over a 10-year period between 1 January 2015 and 31 December 2025. Cases were managed using a multidisciplinary approach involving specialist surgical units including urology, colorectal surgery, and plastic surgery. The patients included in this study provided informed consent for their de-identified medical information to be used.

### Endpoints

The endpoints of interest were related to patient demographics and surgical details, specific timing of and approaches to the management of iatrogenic rectal injury, and postoperative complications.

### Data extraction, synthesis, and statistical analysis

Patient demographic data, including age, sex, indication for radical prostatectomy, prostate cancer stage and grade, prior pelvic radiotherapy, body mass index (BMI), American Society of Anesthesiologists (ASA) grade, and comorbidities, were recorded. Surgical details, including relevant findings within the index operation, details of the rectal injury, timing of and approach to management of the rectal injury, relevant biochemistry or imaging, and any further postoperative complications were also recorded. The collected data were de-identified and stored in a private Microsoft Excel spreadsheet. Heterogeneous or categorical data were reported descriptively. Where relevant, statistical analysis of quantitative data was performed using SPSS Statistics version 25.0 (IBM, Chicago, IL, USA). Results are reported as mean ± standard deviation if normally distributed, or median (interquartile range; IQR) if not normally distributed.

## Results

### Overview of included patients

Over the 10-year period, eight eligible adult men were identified and included in this retrospective consecutive case series. A summary of their demographic data is provided in Table 1. The median age was 67 (IQR 62.5–69.5 years), the median BMI was 25 (IQR 22–27.5), the median ASA score was 2 (IQR 1.5–2.5), and the median Charlson Comorbidity Index (CCI) was 4.5 (IQR 2–6.5). Prostate adenocarcinoma accounted for the majority of cases, whereby a prostatectomy was required (*n* = 6, 75%), with the remainder of cases due to muscle-invasive bladder cancer requiring a cystoprostatectomy with formation of an ileal conduit (*n* = 2, 25%). Tumour grade varied between T2 and T4a disease, with three stage 2 prostate cancer cases, two stage 3 prostate cancer cases, and two stage 3 bladder cancer cases confirmed on postoperative histopathology. For the remaining case, histological details could not be obtained.

**Table 1:** Overview of the included patients.

Patient identifier	Year	Age	Sex	BMI	ASA score	Indication for prostatectomy	Tumour grade	Tumour stage	Prior pelvic radiotherapy	CCI
1	2019	68	M	22	2	High-grade muscle-invasive bladder cancer	T3a	III	Nil	4
2	2020	61	M	23	1	Node-positive prostate adenocarcinoma	T3b	III	Nil	2
3	2021	63	M	27	3	Prostate adenocarcinoma	T2	II	Nil	7
4	2015	70	M	27	3	Prostate adenocarcinoma	T2	II	Nil	8
5	2019	69	M	22	1	Prostate adenocarcinoma	T3b	III	Nil	2
6	2020	62	M	32	2	Prostate adenocarcinoma	NR	NR	Nil	2
7	2023	74	M	28	2	Prostate adenocarcinoma	T2	II	Nil	6
8	2025	66	M	21	2	High-grade muscle-invasive bladder cancer	T4a	III	Nil	5

BMI, body mass index; ASA, American Society of Anaesthesiologists; CCI, Charlson-Comorbidity Index; M, Male; NR, not reported.

### Overview of operative details, identification of rectal injury, and rectal injury management

An overview of operative details and rectal injury profiles is provided in Table 2. Overall, over the 10-year period, 495 prostatectomies were performed with a rectal injury rate of 8/495 (1.62%). Both patients who underwent a cystoprostatectomy and ileal conduit formation had open approach procedures. The remaining radical prostatectomy patients primarily underwent open procedures (*n* = 4, 66%), with the remainder undergoing laparoscopic approaches (*n* = 2, 33%). One case was challenged by significant right-sided pelvic adhesions, and one case was challenged by a significantly enlarged prostate; otherwise, no other significant intraoperative findings that increased the difficulty of the procedures were noted. Rectal injuries occurred in the anterior rectum in all cases and varied from <1 cm to 4 cm. All injuries were full thickness and were either directly observed intraoperatively (*n* = 3, 37.5%) in the immediate setting or via imaging with contrast CT or fluoroscopy in the semi-acute or delayed setting (*n* = 5, 62.5%).

**Table 2:** Operative and rectal injury details.

Patient identifier	Approach to prostatectomy	Additional concurrent procedures	Other finding(s)	Site of rectal injury	Size of defect (cm)	Injury layers	Timing of rectal injury identification	Method of rectal injury identification	Timing of injury repair	Approach to injury repair
1	Open	Cystectomy and ileal conduit formation	Large prostate	Proximal to distal rectum anteriorly	2 × 3	Full thickness	Immediate	Intraoperative visualisation	Immediate	Abdominal approach primary repair in two layers: the seromuscular layer and mucosal layer. No stoma as the tissue and repair quality were deemed good.
2	Open	Bilateral extended lymph node dissection	Nil	Anterior rectum below the peritoneal reflection	3	Full thickness	Semi-acute – 5 days following procedure	CT cystogram	Semi-acute – 5 days	Serial transanal partial closure of lower rectal wall defect with a Penrose drain through the superior half, with re-examination in 2 weeks + a diverting loop sigmoid colostomy.
3	Laparoscopic	Nil	Right- sided pelvic adhesions	Anterior rectum 2–3 cm above anorectal junction	4	Full thickness	Immediate	Intraoperative visualisation	Immediate	Transanal full thickness suture repair + diverting end sigmoid colostomy.
4	Open	Nil	Small prostate	Anterior rectum	2	Full thickness	Immediate	Intraoperative visualisation	Immediate	Abdominal approach primary repair + diverting end sigmoid colostomy.
5	Laparoscopic	Nil	Nil	Anterior rectal fistula due to vesicourethral anastomosis	<1	Full thickness	Delayed – 2.5 years	Methylene blue instilled into the bladder and passed per rectum	Delayed – 2.5 years	Transperineal approach to the excision of the fistula with primary repair of the rectal wall in two layers, i.e., the mucosal layer followed by the seromuscular layer, + closure of the bladder + a left pedicled gracilis flap to interposition the repair between the bladder and the rectal wall + a diverting loop ileostomy.
6	Open	Nil	Nil	Anterior rectum 3 cm above anorectal junction + fistula into the urethra	3	Full thickness	Semi-acute – 11 days	CT followed by intraoperative visualisation	Semi-acute – 11 days then delayed definitive repair – 9 months	Laparotomy and washout + transanal washout of the rectal stump with T-tube insertion into the prostatic cavity and primary closure of the rectal defect + a diverting end sigmoid colostomy. Definitive transperineal repair of the rectourethral fistula with a single-layer closure of the urethra and rectum with interposition of a pedicled left gracilis flap in between.
7	Open	Nil	Nil	Anterior rectum fistulating into posterior urethra/bladder	<1	Full thickness	Semi-acute – 15 days	Fluoroscopic cystogram	Semi-acute – 15 days then delayed definitive repair – 10 months	Diverting end sigmoid colostomy + delayed definitive transperineal repair of rectovesical fistula with single layer closure of bladder and rectum with pedicled left gracilis flap interposition in between
8	Open	Cystectomy and ileal conduit formation	Nil	Anterior rectum x2, left anterior (larger) and right anterior (smaller)	1–2	Full thickness	Semi-acute – 5 days	CT	Semi-acute – 5 days	Initial management with antibiotics and TPN, then proceeded to repair 5 days later via abdominal approach and primary closure + Diverting sigmoid loop colostomy

Repair depended on the site and size of the injury, the quality of the tissue, and the timing of injury identification. Primary abdominal approach repair without stoma formation only occurred in one case. The remainder of cases were repaired transanally with a diverting stoma (*n* = 2, 25%), transperineally with a diverting stoma and gracilis interposition graft (*n* = 3, 37.5%, all for rectovesical fistula), or via an abdominal approach with a diverting stoma (*n* = 1, 12.5%).

### Detailed patient cases

#### Case 1

A 68-year-old man underwent a radical cystoprostatectomy and formation of an ileal conduit due to high-grade, muscle-invasive bladder cancer. During mobilisation of the bladder and prostate away from the rectum, an inadvertent 2 cm × 3 cm rectotomy was created and identified immediately due to the large size of the defect. The injury was primarily repaired in two layers. Tissue strength and quality were deemed adequate with no contamination, and therefore the decision was made to avoid diversion or defunctioning. The patient developed a postoperative ileus, which resolved with conservative management, and was discharged 2 weeks later. He fully recovered and proceeded to adjuvant chemotherapy 6 weeks after his operation. The patient remains asymptomatic and is undergoing ongoing cancer surveillance.

#### Case 2

A 61-year-old man presented with abdominal pain and pyrexia 5 days after an elective open radical prostatectomy. A CT scan of the abdomen and pelvis with portal-venous contrast demonstrated a 6 cm × 4 cm × 10 cm fluid collection in the right iliac fossa. Subsequently, a CT cystogram was performed, which demonstrated the presence of a large rectovesical fistula originating posteriorly from the bladder and communicating with the anterior rectum. An examination under anaesthesia was then performed, which confirmed a 3 cm anterior rectal wall defect that was entirely extraperitoneal. The rectum was irrigated, a drain was inserted into the defect, an indwelling bladder catheter was placed, and a diverting loop sigmoid colostomy was also formed via a left lower quadrant trephine. The patient underwent transanal serial closure 5 days later, during which the lower half of the defect was closed primarily with sutures, and a Penrose drain was placed in the upper half of the defect. A subsequent re-examination 2 weeks later demonstrated near-complete healing; the Penrose drain was subsequently removed, and the patient was discharged. At the 3-month mark, a cystoscopy, cystogram, and a gastrograffin enema study were conducted, demonstrating no persisting rectovesical fistula. The patient underwent elective reversal of the loop sigmoid colostomy 8 months after the stoma was formed and remains well at yearly follow-ups.

#### Case 3

A 63-year-old man was admitted electively for a laparoscopic radical prostatectomy for Gleason 3 + 3 prostate adenocarcinoma. Intraoperatively, a rectal injury was suspected, and the procedure was converted to an open approach. On further inspection, a 4 cm anterior rectotomy was identified 2–3 cm above the anorectal incision from 10 to 1 o’clock; this was deemed too low for a transabdominal repair. A transanal approach was adopted, during which the bruised and traumatised rectotomy edges were excised, and the defect was repaired with interrupted sutures. A rectal washout was performed and an end sigmoid colostomy formed for diversion. The patient was discharged on postoperative day 7. He underwent a reversal of the end sigmoid colostomy 6 months post-procedure with an uncomplicated postoperative course.

#### Case 4

A 70-year-old man underwent an elective open radical prostatectomy. Intraoperatively, a small prostate was identified and, upon mobilisation of it, a 2 cm anterior rectal injury was sustained. The defect was repaired primarily with sutures, and an end sigmoid colostomy was formed for diversion. The patient recovered well and was discharged on day 11 post-operation. He underwent reversal of the end sigmoid colostomy 15 months after the initial procedure and remains well on follow-up.

#### Case 5

A 69-year-old man underwent an elective laparoscopic radical prostatectomy with routine discharge following this procedure. Despite this, the patient reported that he was passing urine per rectum but had no systemic features of sepsis or recurrent urinary tract infection. Multiple flexible cystoscopies and CT cystograms were conducted; however, they failed to demonstrate the presence of any fistula over the course of 2 years. The patient proceeded to a methylene blue test, in which the dye was instilled into the bladder and demonstrated to be passing rectally. The patient subsequently underwent repair of a suspected rectourethral fistula 2.5 years following the date of the initial prostatectomy. A loop ileostomy was fashioned through a trephine incision. Then, through a transperineal approach, the rectum and anus were mobilised away from the urethra and bladder base. The fistula was identified and was repaired using a two-layer closure technique. The defect at the vesicourethral junction was closed with multiple interrupted sutures. Following repair of the fistula tract, a left pedicled gracilis flap was raised and transposed into the defect to interpose the repair between the bladder and the rectal wall. The perineum was closed in layers over a surgical drain. The patient experienced no postoperative recovery complications and was discharged 7 days after his surgical repair. On follow-up, there was resolution of symptoms, and a follow-up sigmoidoscopy revealed no internal opening. The loop ileostomy was reversed after 6 months. The patient remained well at the 12-month mark.

#### Case 6

A 62-year-old man underwent a routine elective open radical prostatectomy and was discharged routinely. He presented 11 days after his initial operation with his midline wound exuding faecal content. A CT of the abdomen and pelvis revealed a potential defect in the anterior rectal wall. The patient underwent an examination under anaesthetic, during which a 3 cm linear defect was identified 3 cm above the anorectal junction. Through this defect, a large cavity extending behind the bladder was found, and an indwelling urinary catheter was palpable at the base of the cavity, suggesting the diagnosis of a rectourethral fistula. A relook laparotomy was performed at this time, which revealed a pre-peritoneal abscess cavity extending downwards into the pelvis behind the bladder containing faecal and purulent material. There was no communication within the peritoneal cavity. This contaminated cavity was extensively irrigated, and a diverting end sigmoid colostomy was created. The defect and the remaining rectal stump were then irrigated transanally, and a T-tube was passed through the defect into the prostatic cavity. The patient underwent primary repair of the rectal defect 10 days later, but the fistula persisted. He then underwent elective repair of the rectourethral fistula 9 months after the index radical prostatectomy. The repair technique was identical to case 5 above, using two-layer repair of the rectum, interrupted sutures for the urethra, and interposition of a left pedicled gracilis flap. The patient made a full recovery. A fluoroscopic cystogram conducted 4 weeks postoperatively demonstrated no leakage of contrast material from the bladder, and the indwelling catheter was removed. His end sigmoid colostomy was reversed 12 months after repair of the rectourethral fistula, and he remains well on surveillance follow-up.

#### Case 7

A 74-year-old man underwent a routine elective open radical prostatectomy. He was discharged after 5 days; however, he presented 15 days later with complaints of passing urine per rectum. A fluoroscopic cystogram was performed, which revealed contrast extravasation, and a subsequent CT demonstrated a connection between the posterior urethra and the anterior rectal wall. The patient underwent creation of an end sigmoid colostomy for diversion and, 10 months later, underwent transperineal repair of the rectourethral/rectovesical fistula. Again, this involved ligation of the fistula tract, primary repair of the bladder/urethra and rectal defects with sutures, and the interposition of a pedicled gracilis flap between the rectum and the bladder, identical to cases 5 and 6. The patient recovered well and was discharged on day 7 after this operation, and his stoma was reversed 10 months later. He remains well on surveillance follow-up.

#### Case 8

A 66-year-old man underwent an elective open radical cystoprostatectomy with formation of an ileal conduit for muscle-invasive bladder cancer after declining neoadjuvant chemotherapy. Over the course of 5 days after the operation, the patient developed what was initially thought to be ileus. This was investigated with a CT scan of the abdomen and pelvis after it was noted that his inflammatory markers were rising (C-reactive protein over 200 mg/mL). The CT scan demonstrated a pneumoperitoneum with findings suggestive of an anterior rectal wall defect. The patient underwent an emergency laparotomy where two intraperitoneal rectal perforations were noted: the larger, 2 cm in length, on the anterior left rectum and the smaller, 1 cm in length, on the anterior right rectum. These defects were closed primarily with sutures, and a leak test was negative. A loop sigmoid colostomy was created for diversion, and the abdominal cavity was lavaged prior to closure. The patient recovered well and underwent a reversal of his loop sigmoid colostomy 6 months later. He remains well on surveillance follow-up.

## Discussion

This retrospective consecutive case series represents one of the few published reports describing the approaches to the management of iatrogenic rectal injury following prostatectomy, with a focus on a regional setting. With a decade of data, our series of eight patients highlights the clinical rarity of this complication but also demonstrates that successful patient outcomes are achievable through early recognition and multidisciplinary surgical decision-making. When considering these injuries, the literature suggests prior pelvic radiotherapy, prostatectomy at low-volume centres, and the extent of the prostate tumour (grade and stage) as potential contributors (5, 8, 9). We did not observe these risk factors, nor the presence of significant pelvic adhesions, in the majority of the included cases. This highlights that these injuries, although unfortunate and rare, may occur in patients despite the absence of significant risk factors. Despite this, these lower-risk patient profiles may have contributed to the favourable tissue quality observed at repair that allowed primary suture closure of the defect; this was best exemplified in case 1 where no diversion was required. Additionally, all the patients who were diverted were reversed within a year of their stoma being created. Notably, our patient cohort was quite young, with a median age of 67, and from a comorbidity and ASA perspective, with subjects who were fit and well, reflecting a greater physiological reserve. These factors may explain the observed successful outcomes and lack of complications identified following rectal injury repair in our study.

To date, there remains a lack of standardised guidelines for how these rectal injuries should be managed (5). This is particularly relevant in regional settings, where the specialist colorectal, plastic surgery, or urology expertise to treat deep pelvic injuries may be limited or non-existent. Our cases highlight that a critical determinant of management and outcomes was the timing of injury identification. Of the three injuries identified intraoperatively, all were repaired immediately by primary closure of the defect with or without diversion. Despite this, the majority of the cases (*n* = 5, 62.5%) were identified sub-acutely or in a rather delayed manner, ranging from days to years, with the additional modalities required including imaging, examination under anaesthesia, and methylene blue testing. This finding aligns with the existing literature that demonstrates a large proportion of rectal injuries are not recognised at the time of the index operation, particularly as some of these cases are due to small defects caused by traction or devascularisation rather than direct laceration or surgical trauma (5). In this sense, they manifest later as ischaemia progresses towards full-thickness perforation of the rectum in these delayed cases. The consequences of delayed recognition were evident in this series, with three cases developing rectovesical or rectourethral fistulae requiring radiological diagnosis with fluoroscopy or contrast CT and one case requiring dye testing. This was then followed by multi-stage surgical repair, diversion, and perineal dissection with interposition of pedicled gracilis flaps. Additionally, these patients also presented with persistent symptoms that impacted their quality of life and therefore had unrecognised morbidity in their everyday life following surgery. Therefore, it is necessary that, in regional surgical centres where a prostatectomy service is available, mechanisms are in place to streamline assessment of potential rectal injury when suspicion is present to ensure timely diagnosis and management.

When considering the repair strategy, our varied approaches considered defect size and location, presence of contamination, timing of injury identification, tissue quality, and the presence of an established fistula. In cases of immediate recognition, such as in case 1, excellent tissue quality, a small defect size, and a lack of contamination may allow for successful primary repair without diversion. In contrast, for injuries where friable tissue is involved or those that are identified later, complexity arises whereby there is a greater need to consider the role of diversion and whether an abdominal, perineal, or transanal approach should be considered. In such cases, the experience of the available surgeons, particularly regarding these operative rescue repair options, should be considered. In a regional service performing a high volume of prostatectomies, healthcare service providers should consider adequate training or acquisition of specialist colorectal and plastic surgery expertise to provide care for these patients. This is particularly important for timely diagnosis and surgical management. Finally, the management of established rectovesical and rectourethral fistulae warrants consideration. All three cases (cases 5–7) underwent staged management with faecal diversion followed by transperineal repair with interposition of a pedicled gracilis flap. This transperineal approach allows broader anatomical access to both the urethra and anterior rectum, enabling precise ligation of the fistula tract, primary repair of the involved structures, and adequate separation of tissue planes (3, 4). The gracilis flap, in particular, has been shown to improve fistula closure rates, which highlights the role of specialist plastic surgical units in the management of rectal injuries following prostatectomy (7).

**Figure 1:**
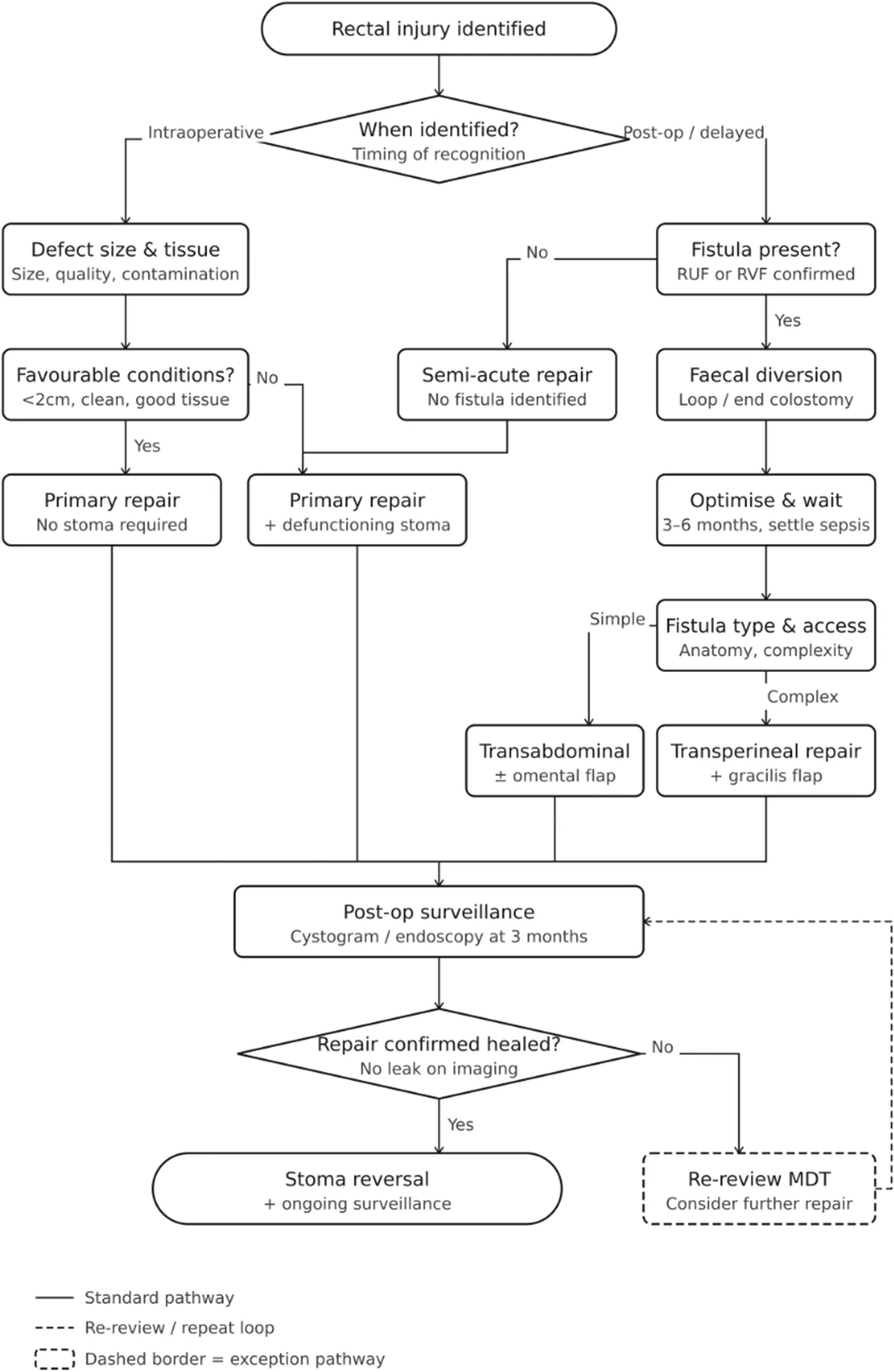
Proposed algorithm for the management of rectal injury following prostatectomy. RUF, rectourethral fistula; RVF, rectovesical fistula; MDT, multidisciplinary team.

Based on our experience, we propose a structured management algorithm to assist regional institutions in decision-making and refining the multidisciplinary approach to the management of these injuries. The proposed algorithm stratifies decision-making based on relevant domains that we found affected patient outcomes, such as the timing of injury identification and the size of the injury (Figure 1). In the first domain, patients with an intraoperatively recognised small rectal injury (<2 cm) that occurs in the absence of prior pelvic irradiation, established urethral stricture, or significant contamination are considered candidates for primary repair without faecal diversion. Where the defect is larger, the tissue quality is suboptimal, or the injury is identified in the immediate postoperative period before the onset of severe sepsis, primary repair with a defunctioning/diverting stoma is recommended. For patients in whom the injury is identified following hospital discharge or where an established fistula is confirmed, initial management with faecal diversion is recommended, followed by delayed definitive repair at 3–6 months once the operative field has been optimised. In this context, transperineal repair with interposition of a pedicled gracilis flap is the preferred approach for rectourethral and rectovesical fistulae; however, the literature also suggests a transabdominal repair with interposition of an omental patch for simpler fistulae. Finally, surveillance to assess the repair should be conducted, such as with a radiological cystogram and endoscopy at 3 months, before reversal of the stoma is considered. The relevance of this algorithm to the regional and rural Australian (and arguably any developed country across the world) surgical workforce is particularly pertinent given current and projected trends in prostate cancer. As Australia's population ages and retirement migration to non-metropolitan regions increases, a growing proportion of radical prostatectomies will be performed in regional centres. However, the opportunity to identify these injuries proactively should also be considered. For example, conducting a leak test following prostatectomy, such as air or a diluted povidone-iodine solution inserted per rectum, may be an effective and low-cost approach for routine exclusion of a rectal injury before closure. Finally, it is important to consider the limitations of this study, which predominantly stem from the small sample size. Specifically, given the non-normal distribution, the performed inferential statistical analyses should be interpreted carefully, and the generalisability of these results should be considered with caution. Overall, our experience highlights that these more complicated urological operations will generally occur at centres with good colorectal and plastic surgery support, highlighting the need for synergistic cross-speciality cooperation for the timely and effective diagnosis and management of rectal injuries following prostatectomy.

## Conclusion

A rectal injury following radical prostatectomy is a rare but serious complication. It demands prompt recognition, structured multidisciplinary management, and surgical decision-making tailored to the timing, severity, and context of the injury. Our 10-year regional experience of these injuries demonstrates that this complication can be managed with good patient outcomes through a combination of intraoperative vigilance, early postoperative investigation, appropriate faecal diversion, and staged definitive repair when required. The proposed management algorithm and these positive outcomes, however, require diverse and well-trained surgical teams that are equipped to manage deep pelvic injuries. Therefore, we advocate for the upskilling of the regional surgical workforce in this regard as prostate cancer care in regional centres becomes more established.

## Data Availability

The original contributions presented in the study are included in the article/Supplementary Material, further inquiries can be directed to the corresponding author.
